# *Pcsk5* is required in the early cranio-cardiac mesoderm for heart development

**DOI:** 10.1186/s12861-017-0148-y

**Published:** 2017-04-26

**Authors:** Dorota Szumska, Milena Cioroch, Angela Keeling, Annik Prat, Nabil G. Seidah, Shoumo Bhattacharya

**Affiliations:** 1 Division of Cardiovascular Medicine, Radcliffe Department of Medicine, University of Oxford, the Wellcome Trust Centre for Human Genetics, Roosevelt Drive, Oxford, OX3 7BN UK; 2 Laboratory of Biochemical Neuroendocrinology, Montreal Clinical Research Institute (IRCM), 110 Pine Ave west, Montreal, QC H2W1R7 Canada

**Keywords:** Cardiogenesis, *Pcsk5*, Mouse, Conditional knock-out

## Abstract

**Background:**

Loss of proprotein convertase subtilisin/kexin type 5 (*Pcsk5*) results in multiple developmental anomalies including cardiac malformations, caudal regression, pre-sacral mass, renal agenesis, anteroposterior patterning defects, and tracheo-oesophageal and anorectal malformations, and is a model for VACTERL/caudal regression/Currarino syndromes (VACTERL association - Vertebral anomalies, Anal atresia, Cardiac defects, Tracheoesophageal fistula and/or Esophageal atresia, Renal & Radial anomalies and Limb defects).

**Results:**

Using magnetic resonance imaging (MRI), we examined heart development in mouse embryos with zygotic and cardiac specific deletion of *Pcsk5*. We show that conditional deletion of *Pcsk5* in all epiblastic lineages recapitulates all developmental malformations except for tracheo-esophageal malformations. Using a conditional deletion strategy, we find that there is an essential and specific requirement for *Pcsk5* in the cranio-cardiac mesoderm for cardiogenesis, but not for conotruncal septation or any other aspect of embryonic development. Surprisingly, deletion of *Pcsk5* in cardiogenic or pharyngeal mesodermal progenitors that form later from the cranio-cardiac mesoderm does not affect heart development. Neither is *Pcsk5* essential in the neural crest, which drives conotruncal septation.

**Conclusions:**

Our results suggest that *Pcsk5* may have an essential and early role in the cranio-cardiac mesoderm for heart development. Alternatively, it is possible that *Pcsk5* may still play a critical role in *Nkx2.5*-expressing cardiac progenitors, with persistence of mRNA or protein accounting for the lack of effect of deletion on heart development.

**Electronic supplementary material:**

The online version of this article (doi:10.1186/s12861-017-0148-y) contains supplementary material, which is available to authorized users.

## Background

We have previously described a complex developmental phenotype in mice bearing a homozygous ethylnitrosourea-induced mutation (*Vcc*) or a conditional epiblastic knockout of the gene encoding the proprotein convertase subtilisin/kexin type 5 - *Pcsk5* [1, 2]. No phenotypic abnormalities were observed in heterozygous mice. Components of the homozygous or epiblastic knockout mutant phenotype included cardiac malformations such as dextrocardia, atrial and ventricular septal defects (ASD, VSD), common arterial trunk (CAT), vascular ring, right-aortic arch and hypoplastic arterial duct. Such lesions are characteristic for human congenital heart disease (CHD) [3] – a gross structural abnormality of the heart or intrathoracic great vessels that is present at birth and is of functional significance [4]. *Pcsk5* mutation also resulted in non-cardiac defects commonly associated with CHD: antero-posterior patterning defects, tracheo-esophageal and anorectal malformations, presacral mass, absent tail, sacral agenesis, increased numbers of thoracic vertebrae and true ribs, hypoplastic hind limbs, renal and palatal agenesis, and pulmonary hypoplasia. Together with CHD-like malformations, this phenotype closely resembles human VACTERL/caudal regression/Currarino syndrome-like malformations (respectively, OMIM192350, 600145, 176450). We [1] and others [5] have found heterozygous mutations in conserved residues in *PCSK5* in patients with VACTERL syndrome that are transmitted from phenotypically normal parents, suggesting that other genetic or epigenetic factors that interact with *PCSK5* are likely responsible for the development of the disease.

PCSK5 is a member of the subtilisin-like proprotein convertase family that mediates pro-domain cleavage and activation of TGFβ/BMP-family members (Transforming growth factor beta / Bone morphogenetic protein family) [6]. *Pcsk5* is expressed in extraembryonic lineages in the mouse at embryonic day (E) 6.5 [2], and then expression initiates in the anterior mesoderm of the embryo at around E7.5, just before the cardiac crescent is formed [7]. At E9.5 *Pcsk5* is expressed in the somites, bulb of umbilical cord and lung buds [8]. We have previously shown, using whole mount *in situ* hybridisation at E10.5, that *Pcsk5* is present in the somites and limb buds but we did not observe it in the heart or outflow tract [1]. Using a more sensitive radioactive *in situ* hybridisation method, we have shown previously that at E10.5-E11.5 *Pcsk5* is, indeed, weakly expressed in the *bulbus cordis* [8]. We have previously shown that deletion of *Pcsk5* (exon 1 and exon 4 deletions, referred to as *Δ1* and *Δ4*) led, respectively, to pre-natal or early embryonic lethality [2, 8]. By characterizing the *Vcc* and conditional epiblastic *Δ1* mutations, we showed that *Pcsk5* functions to cleave and activate GDF11 (Growth differentiation factor 11), and thus regulates caudal *Hox* paralogs (Homeobox genes) to control anorectal, renal, and caudal skeletal development [1, 2]. The presence of cardiac abnormalities in the *Vcc* and conditional epiblastic *Δ1* mutations indicated that PCSK5 is essential for heart development, but these studies did not address the exact spatio-temporal requirements of this enzyme during cardiogenesis. Cardiac malformations arise from defects in progenitor cell specialisation, or abnormal patterning during development. The heart, although induced by endodermal signals, is essentially mesodermal in origin, with a minor contribution from the ectodermal neural crest [9]. These three embryonic lineages arise from the epiblast [10]. Following gastrulation, mesodermal progenitors migrate anteriorly to form two closely appositioned primary and secondary heart fields (E7-7.5) [11–14]. These cells specialize into cardiomyocyte, endothelial and epicardial lineages that form the heart. Neural crest cells differentiate into the mesenchyme of the great arteries and transiently contribute to the outflow tract (cono-truncal) and aorticopulmonary septa [15, 16]. Left-right patterning also plays a key role in creating a 4-chambered heart [9, 11]. The heart tube is initially linear (E7.5-8), with venous tributaries draining into the developing atria at the posterior or inflow end, and connecting to the ventral aorta at its anterior or outflow end. The initially symmetrical atria subsequently develop distinct left-right identities. The heart tube undergoes dextral looping beginning E8.25 and remodelling between E10.5–12.5, processes that are necessary to position the developing atria cranial to the ventricles, and to connect the left and right atria to the respective ventricles [11]. The aorta, carotid and pulmonary arteries arise by remodelling of the initially bilateral pharyngeal arterial arch system, beginning at E11.5 [17]. By E15.0, major structures of mouse embryonic heart are fully developed; maturation of ventricular and atrial septa, separation of outflow tract and cardiac valve formation is completed [18]. To explore further the role of PCSK5 during heart development, we employed a conditional knockout approach to ablate *Pcsk5* from cardiogenic lineages. We show that conditional deletion of *Pcsk5* in the early cranio-cardiac mesoderm affects heart development but that conditional deletion in the neural crest or in the pharyngeal arches, or in *Nkx2.5*-expressing cardiac progenitors does not affect heart development.

## Methods

### Mice and generation of embryos

All animal procedures were approved by the Committee for Animal Care and Ethical Review at the University of Oxford, and all the experiments conformed to the UK Animals (Scientific Procedures) Act, 1986, incorporating Directive 2010/63/EU of the European Parliament. Mice were housed in groups, in specific pathogen-free cages under a 12 h light-dark cycle, at 21–22 °C, with chow and drinking water available *ad libitum* (Teklad global 16% rodent diet, 2916, Harlan UK). Mice with *Pcsk5* alleles *Pcsk5* ^*tm2.1Prat*^ (referred to as *Δ1*) and *Pcsk5* ^*tm2Prat*^ (referred to as *Pcsk5* ^*flox*^) have been previously described [2]. *Pcsk5* ^*flox*^ has the proximal promoter and exon 1 framed by *loxP* sites and the *Δ1 KO* allele has this fragment permanently deleted. *Psck5* ^*Δ1/Δ1*^ embryos were generated by intercrossing *Pcsk5* ^*+/Δ1*^ animals. Conditional deletion of *Pcsk5* was achieved by crossing *Pcsk5* ^*+/Δ1*^ mice with *Sox2Cre* (Tg(Sox2-cre)1Amc) [19], *Mesp1Cre* (*Mesp1* ^*tm2(cre)Ysa*^) [20], *Nkx2.5Cre* (*Nkx2-5* ^*tm1(cre)Rjs*^) [21], *Hoxa3Cre* (*Hoxa3* ^*tm1(cre)Moon*^) [22] and *Wnt1Cre* (Tg(Wnt1-cre)11Rth) [15] to generate mice with *Pcsk5* ^*Δ1/flox*^ *; Cre* ^*+*^ genotypes. To avoid general recombination due to *Cre* expression in the maternal germline [23], only males were used as a source of *Cre* recombinase. First, we created double heterozygous males by crossing *Pcsk5* ^+/Δ1^ animals with a *Cre* line. Selected males (*Pcsk5* ^*+/Δ1*^ *; Cre* ^*+*^) were then used to generate *Pcsk5* ^*flox/Δ1*^ *; Cre* ^*+*^ embryos by crossing to *Pcsk5* ^*flox/flox*^ females. Pregnant dams were killed by cervical dislocation and the embryos were dissected and processed for further analyses as described below. Genotyping was performed using polymerase chain reaction with allele-specific primers (details available on request) on DNA isolated from embryonic forelimbs or from sections of cardiac tissue.

### Magnetic resonance imaging (MRI)

Embryos were dissected at indicated time-points and MRI was performed and data analysed as described previously [1, 24].

### Histology

Embryos were dissected into cold PBS (Phosphate-buffered saline) at various developmental stages and fixed in 4% paraformaldehyde. Histological sections and hematoxylin–eosin staining were performed as described previously [1, 25].

### In situ hybridization

*In situ* hybridization was performed using a digoxigenin-labeled antisense probe as described previously [1].

## Results and discussion

### Pcsk5 expression in early murine embryos

**Fig. 1:**
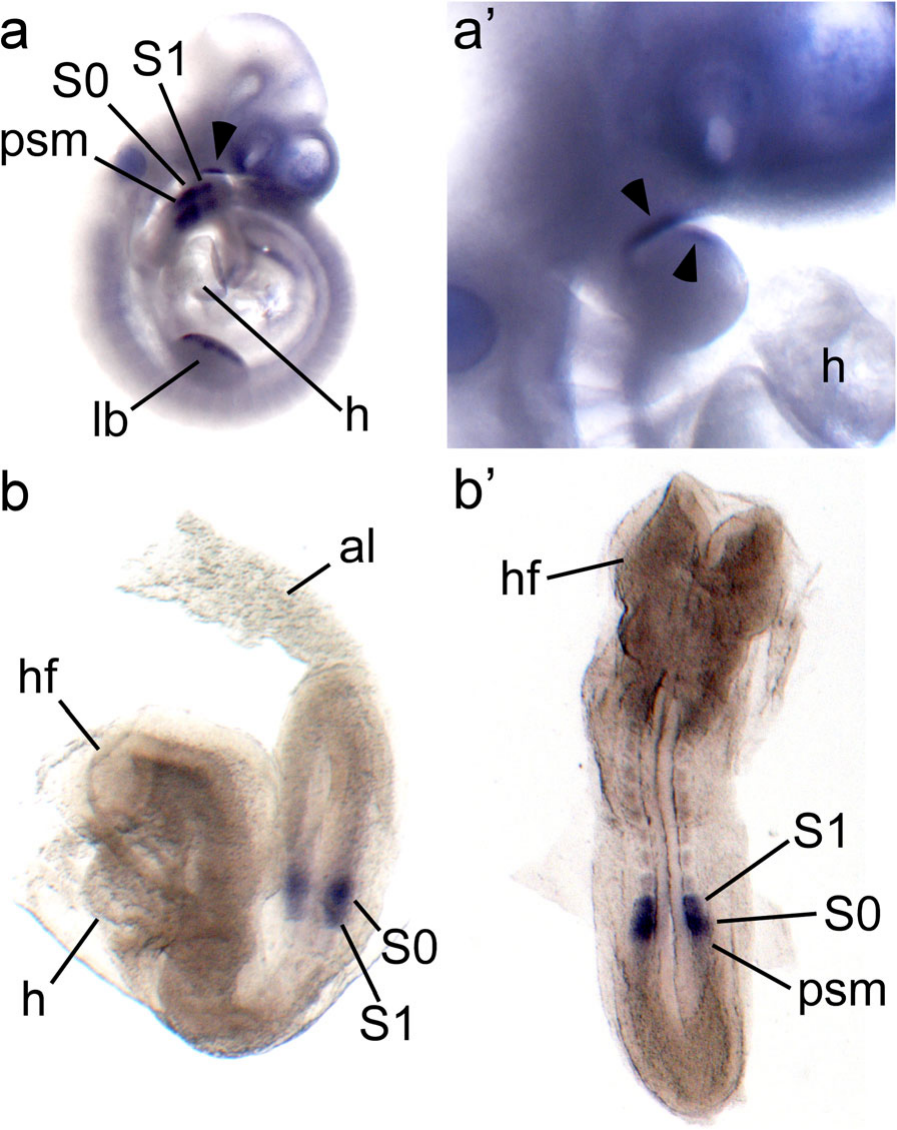
*Pcsk5 e*xpression in the wild-type embryos. *Pcsk5* gene expression analysis using *in situ* hybridization. (**a**) Lateral view of an E10.5 wild-type embryo and a close up of its pharyngeal arches (*a’*). Strong staining is visible in the limb bud (*lb*), presomitic mesoderm (*pms*), forming (*S0*) and last formed (*S1*) somites, and in the pharyngeal arch (*black arrowheads* in *a* and *a’*). Heart (*h*) is indicated. (**b**) Lateral and (*b’*) dorsal views of an E8.5 wild-type embryo showing strong staining only in forming and last formed somites (*S0*, *S1*) and a weak expression in presomitic mesoderm (*pms*) but not in the heart tube (*h*) or structures surrounding the heart. Head fold (*hf*) and allantois (*al*) are indicated

To further characterize the expression of *Pcsk5* we used whole mount *in situ* hybridisation. We find while *Pcsk5* is expressed in the presomitic mesoderm and first forming somites at E8.5 (Fig. 1), it is not seen in the forming heart tube. *Pcsk5* is also detected in the pharyngeal arches at E10.5 (Fig. 1). Taken together with published data this suggests that with regard to heart, outflow tract and aortic arch development, *Pcsk5* could have either an early role in the developing mesoderm, or a later role in the *bulbus cordis* (which forms the outflow tract of the heart) or the pharyngeal arches.

### Pcsk5 Δ1 knock-out embryos recapitulate completely the Vcc mutation

We used magnetic resonance imaging (MRI) to analyse *Pcsk5* ^*Δ1/Δ1*^ knock-out embryos at E15.5. Screening embryos at this developmental stage allows the observation of cardiac malformations that are typical of human congenital heart disease. We imaged 4 *Pcsk5* ^*Δ1/Δ1*^, 10 heterozygous (*Pcsk5* ^Δ1/wt^) and 3 wild-type embryos (*Pcsk5* ^wt/wt^). All four *Pcsk5* ^*Δ1/Δ1*^ embryos had reduced body size, caudal regression evidenced by hind limb dysplasia and absent tail, visceral anomalies including exomphalos, absent kidneys, neural presacral mass, hypoplastic lungs, tracheo-esophageal fusion, and cardiac malformations. These included ventricular and atrial septal defects, abnormal atrio-ventricular junction, common arterial trunk, and right-sided aortic arch. These abnormalities were not found in heterozygous and wild-type littermates (Table 1). Thus the *Pcsk5* ^*Δ1/Δ1*^ knock-out fully recapitulates the *Vcc* mutant allele.

**Table 1:** Developmental anomalies identified by MRI in *Pcsk5* zygotic and conditional knock-out embryos at E15.5

Phenotype	MP_term	*Pcsk5* ^*Δ1/Δ1*^	*Pcsk5* ^*Δ1/flox*^ *; Sox2Cre+*	*Pcsk5* ^*Δ1/flox*^ *; MespI1Cre+*	*Pcsk5* ^*Δ1/flox*^ *; Nkx2.5Cre+*	*Pcsk5* ^*Δ1/flox*^ *; Hoxa3Cre+*	*Pcsk5* ^*Δ1/flox*^ *; Wnt1Cre+*
		*n* = 4 (1)	*n* = 5 (1)	*n* = 6 (3)	*n* = 6 (5)	*n* = 5 (2)	*n* = 8 (5)
reduced body size	MP:0001698	4	5	–	–	–	–
oedema	MP:0001785	4	5	4	–	–	–
cleft palate	MP:0000111	4	5	4	–	–	–
tracheo-esophageal fusion	MP:0003117	3	–	–	–	–	–
exomphalos	MP:0003052	4	5	–	–	–	–
abnormal rectum	MP:0000492	4	5	–	–	–	–
absent kidneys	MP:0000520	4	5	–	–	–	–
abnormal lungs	MP:0003641 MP:0001175	4	5	–	–	–	–
neural presacral mass	MP:0000955	4	4	–	–	–	–
absent tail	MP:0003456	4	5	–	–	–	–
hind limb dysplasia	MP:0000556	4	5	–	–	–	–
ASD	MP:0000282	4	4	1	–	–	–
VSD	MP:0000281	4	5	4	–	–	–
abnormal AVJ	MP:0006197	4	5	6	–	–	–
DORV	MP:0000284	–	2	3	–	–	–
TGA	MP:0006127	–	1	1	–	–	–
CAT	MP:0002633	4	1	–	–	–	–
R-AoA	MP:0004158	1	2	–	–	–	–
R-DA	MP:0000486	–	1	–	–	–	–
IAA	MP:0004157	–	1	–	–	–	–

Developmental anomalies observed in *Pcsk5* ^*Δ1/Δ1*^ knock-out embryos and embryos with conditional deletion of *Pcsk5* in cardiac lineages (*Pcsk5* ^*Δ1/flox*^ *Cre+).* The number of embryos analysed for each group (n) is indicated, and the number of independent litters these embryos came from is indicated in parentheses. Mammalian Phenotype terms (MP_term) are shown for each anomaly observed. *ASD* atrial septal defect, *VSD* ventricular septal defect, *AVJ* atrio-ventricular junction, *DORV* double outlet right ventricle, *TGA* transposition of great arteries, *CAT* common arterial trunk, *R-AoA* right-sided aortic arch, *R-DA* right-sided ductus arteriosus, *IAA* interrupted aortic arch

### Epiblast deletion of Pcsk5 almost completely recapitulates the zygotic mutant [26] phenotype

**Fig. 2:**
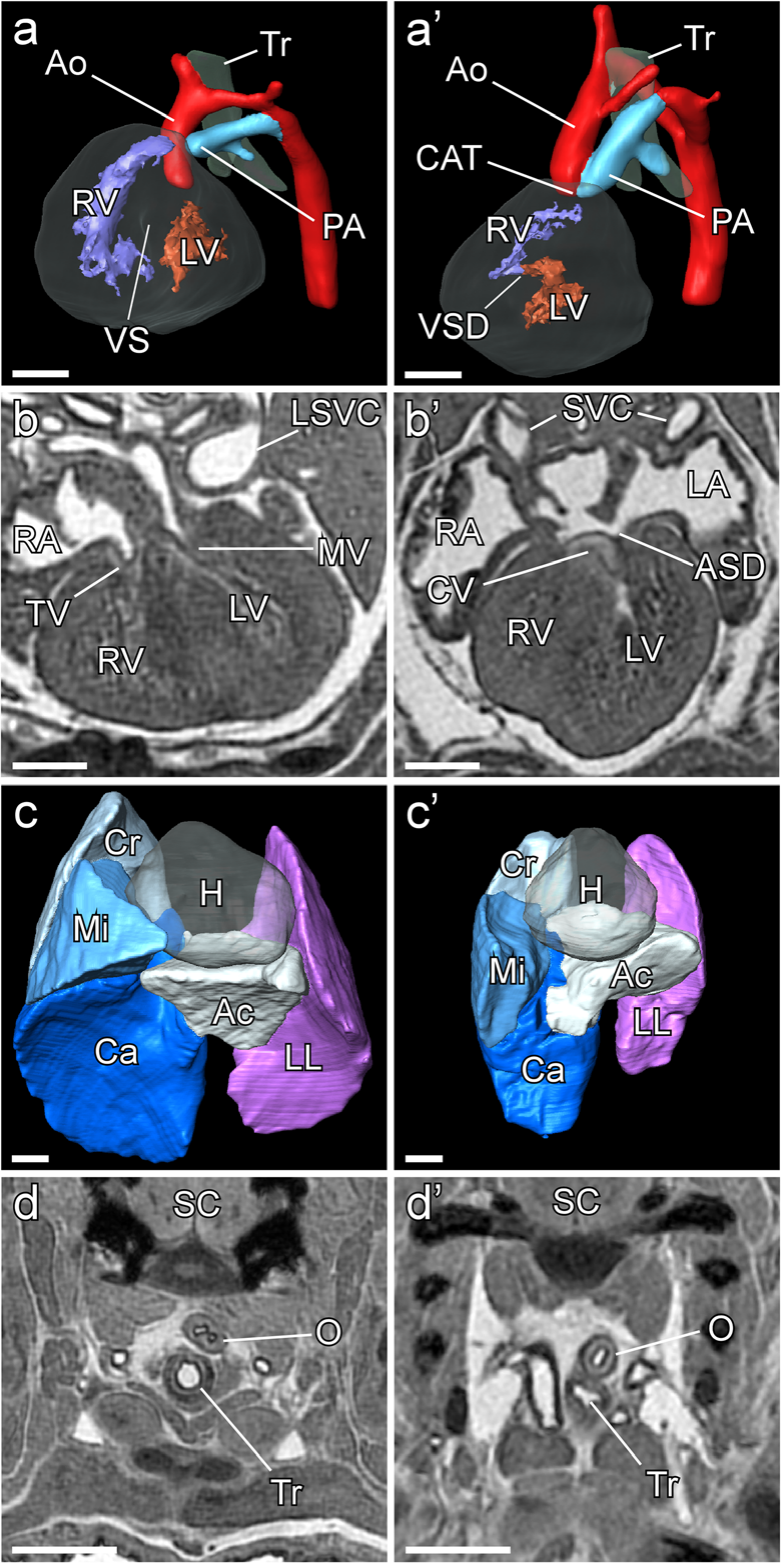
Cardiovascular and pulmonary anomalies in the *Pcsk5; Sox2Cre* conditional KO embryos. (**a**) Three-dimensional reconstruction of the control, *Pcsk5* ^*wt/flox*^ *; Sox2Cre +* heart at E15.5. At this stage, ventricular septum (*VS*) is fully formed, aorta (*Ao*) leaves left ventricle (*LV*), and pulmonary artery (*PA*) leaves right ventricle (*RV*). Both great arteries go to the left side of the trachea (*Tr*); (*a’*) *Psck5* ^*Δ1/flox*^; *Sox2Cre* heart showing ventricular septal defect (*VSD*), common arterial trunk (*CAT*) and aorta forming right-sided arch. Pulmonary artery (*PA*) joins the aorta on the correct, left side of the trachea. (**b**) MRI sagittal section through control heart with tricuspid and mitral valves (*TV*, *MV*) and a correct septation. (*b’*) Corresponding section through the *Psck5* ^*Δ1/flox*^; *Sox2Cre* embryo with atrial septal defect (*ASD*) and common, thickened atrioventricular valve (*CV*); on both sections, left and right ventricles (*LV*, *RV*) and right and / or left atria (*RA*, *LA*) are shown, as well as superior venae cavae (*SVC*, *LSVC* – left *SVC*). (*c*, *c*’) 3D reconstructions of normal and conditional knock-out) cKO lungs; both embryos have three pulmonary lobes on the right side (*Cr* – cranial, Mi – middle, *Ca* – caudal lobe) and accessory lobe (*Ac*) extending to the left side. Left lung (*LL*) has only one lobe. Heart position (*H*) is shown. *Psck5* ^*Δ1/flox*^ *; Sox2Cre* embryo (*c’*) has clearly reduced lungs’ size in comparison to its normal littermate (**c**). *Psck5* ^*Δ1/flox*^; *Sox2Cre* embryos do not have a tracheo-oesophageal fusion as it was observed in a *Vcc* and zygotic mutants. Both control embryo (**d**) and *Psck5* ^*Δ1/flox*^; *Sox2Cre* littermate (*d’*) have trachea (*Tr*) and oesophagus (*O*) properly separated, as shown on this MRI sagittal sections. All scale bars = 0.5 mm

**Fig. 3:**
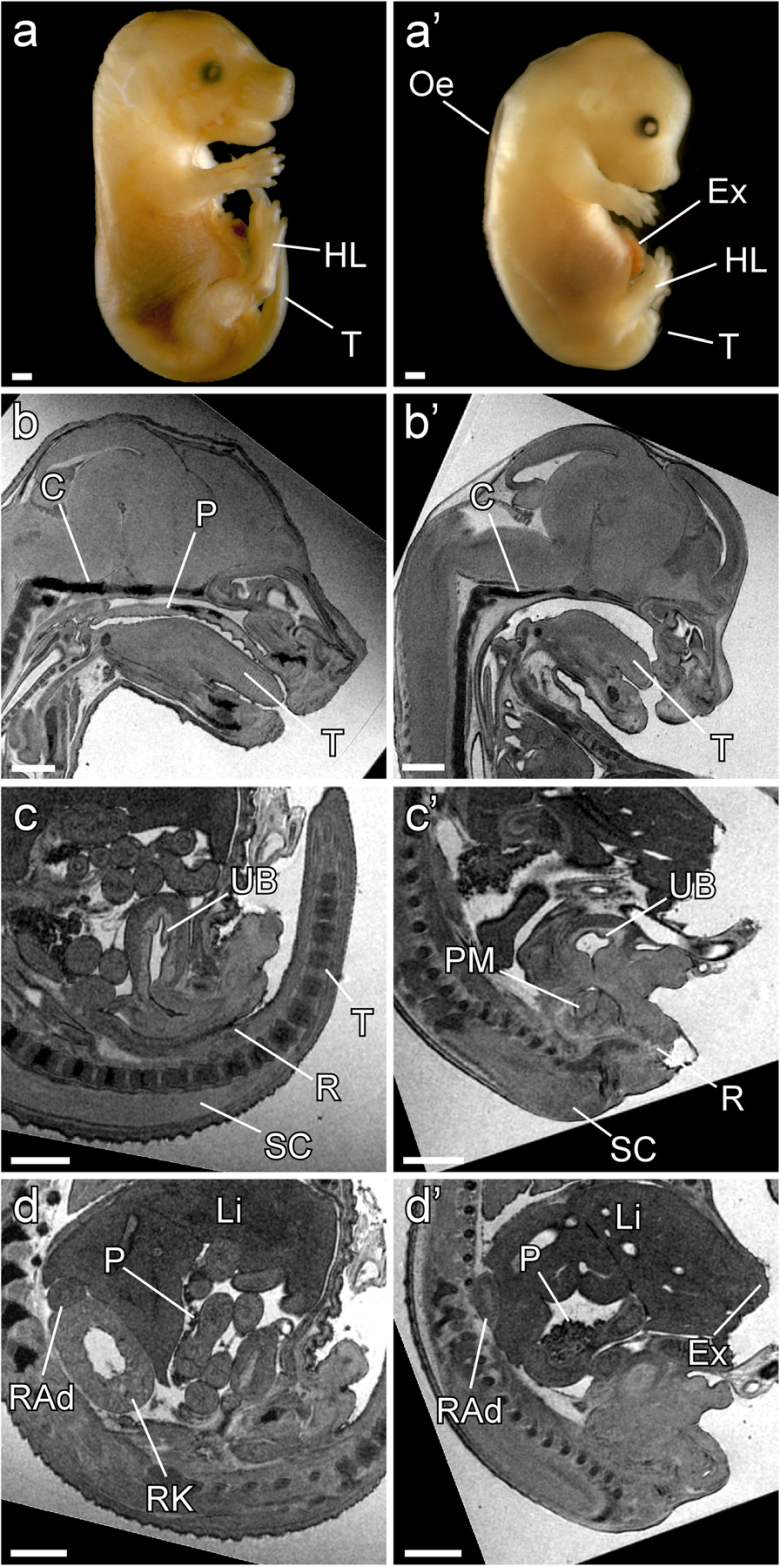
External appearance, palatal and visceral anomalies in the *Pcsk5; Sox2Cre* conditional KO embryos. (**a**) External appearance of a control and *Psck5* ^*Δ1/flox*^; *Sox2Cre* (*a*’) embryos at E15.5. (*a’*) *Psck5* ^*Δ1/flox*^; *Sox2Cre* embryo is smaller than its control littermate, has oedema (*Oe*), hypoplastic hind limbs (*HL*) and exomphalos (*Ex*). The tail (*T*) is absent. (*b’*) MRI transverse section showing large palatal cleft in a *Psck5* ^*Δ1/flox*^; *Sox2Cre* embryo in comparison to its control littermate’s fully developed palate (*P*) (**b**). Tongue (*T*) and clivus (*C*) are shown. (*c’*) MRI transverse section through the *Psck5* ^*Δ1/flox*^; *Sox2Cre* embryo. The urinary bladder (*UB*) and the rectum (*R*) are disrupted by the presacral mass (*PM*). Spinal cord (*SC*) does not extend into the tail. (**c**) Correctly formed urinary bladder (*UB*), rectum (*R*) and tail (*T*) in a control embryo. (*d’*) *Psck5* ^*Δ1/flox*^; *Sox2Cre* embryo lacks kidneys although adrenal glands are presents; right adrenal gland (*RAd*), pancreas (*P*) and liver (*Li*) are indicated in the picture as well as right kidney (*RK*) in the control littermate (**d**). All scale bars = 0.5 mm

Cardiac developmental malformations can result from abnormalities in extra-embryonic lineages that affect placental development [27]. To distinguish the role of *Pcsk5* in the extraembryonic versus embryonic lineages we have previously reported epiblastic deletion of *Pcsk5* with *Meox2Cre*, and found that this recapitulated all developmental malformations but with reduced penetrance [1]. We therefore used a *Sox2Cre* driver that also deletes mainly in the epiblast [19] (Table 2) to explore if a different driver would affect penetrance. Although *Sox2* is expressed in the trophoblast extraembryonic lineage [28], reports show, that *Sox2Cre*-dependent deletion does not significantly affect extraembryonic tissues [29, 30]. We generated and analysed five *Pcsk5* ^*Δ1/flox*^ *; Sox2Cre +* embryos and four *Pcsk5* ^*wt/flox*^ *; Sox2Cre +* control littermates. While all control embryos were normal, *Pcsk5* ^*Δ1/flox*^ *; Sox2Cre +* embryos had anomalies observed previously in the *Vcc* and *Δ1* mutants (Table 1). Cardiac anomalies were present in all *Pcsk5* ^*Δ1/flox*^ *; Sox2Cre +* embryos and included: ventricular and atrial septal defects, abnormal atrio-ventricular junction, common arterial trunk, and double outlet right ventricle, right-sided aortic arch, and interrupted aortic arch (Fig. 2, Table 1). Non-cardiac malformations included hypoplastic lungs, palatal cleft, exomphalos, absent kidneys, presacral mass and skeletal abnormalities that included small hind limbs, and absent tail (Fig. 3, Table 1). We did not observe tracheo-oesophageal fusion, which was present in both the *Pcsk5* zygotic knockout and *Vcc* alleles, in any of the embryos with epiblast deletion of *Pcsk5* (Fig. 2, Table 1). Taken together these results indicated that all embryonic malformations observed in the zygotic mutations arise from a requirement of *Pcsk5* in lineages derived from the epiblast, which includes the mesoderm, endoderm, ectoderm and neural crest.

**Table 2:** Tissue-specific Cre recombinase drivers and their relationship to cardiovascular lineages

Cre driver	Time of deletion [E]	Tissue	Cardiac and vascular derivatives at E14.5 - E15.5
Sox2Cre [19]	6.5	Epiblast	All embryonic tissues.
Wnt1Cre [15, 51]	8.5	Neural crest	Aorticopulmonary septum, conotruncal cushions, 3^rd^, 4^th^ and 6^th^ pharyngeal arch arteries.
Hoxa3Cre [22, 44, 52]	E8.0 - E9.0	Pharyngeal arch lineages (endo, meso and ectoderm), caudal to the second arch (PA 3–6).	Pharyngeal arch arteries (smooth muscle cells), myocardium of a distal outflow tract and the base of the pulmonary trunk, outflow tract cushions.
Mesp1Cre [31–33]	6.5–7.0	Extraembryonic, lateral, cranio-cardiac, and pharyngeal mesoderm.	Myo-, endo- and epicardium, pharyngeal arch arteries.
Nkx2.5Cre [21, 36, 38]	7.5–7.75	Myocardial precursors of primary and secondary heart fields, pharyngeal endoderm and ectoderm of the first pharyngeal arch, proepicardium.	Myocardium; endothelium of coronary arteries, cardiac chambers, and valves; smooth muscle cells of the aorta and coronary arteries.

### Conditional deletion of Pcsk5 in early cranio-cardiac mesoderm recapitulates cardiac anomalies observed in the zygotic mutations

**Fig. 4:**
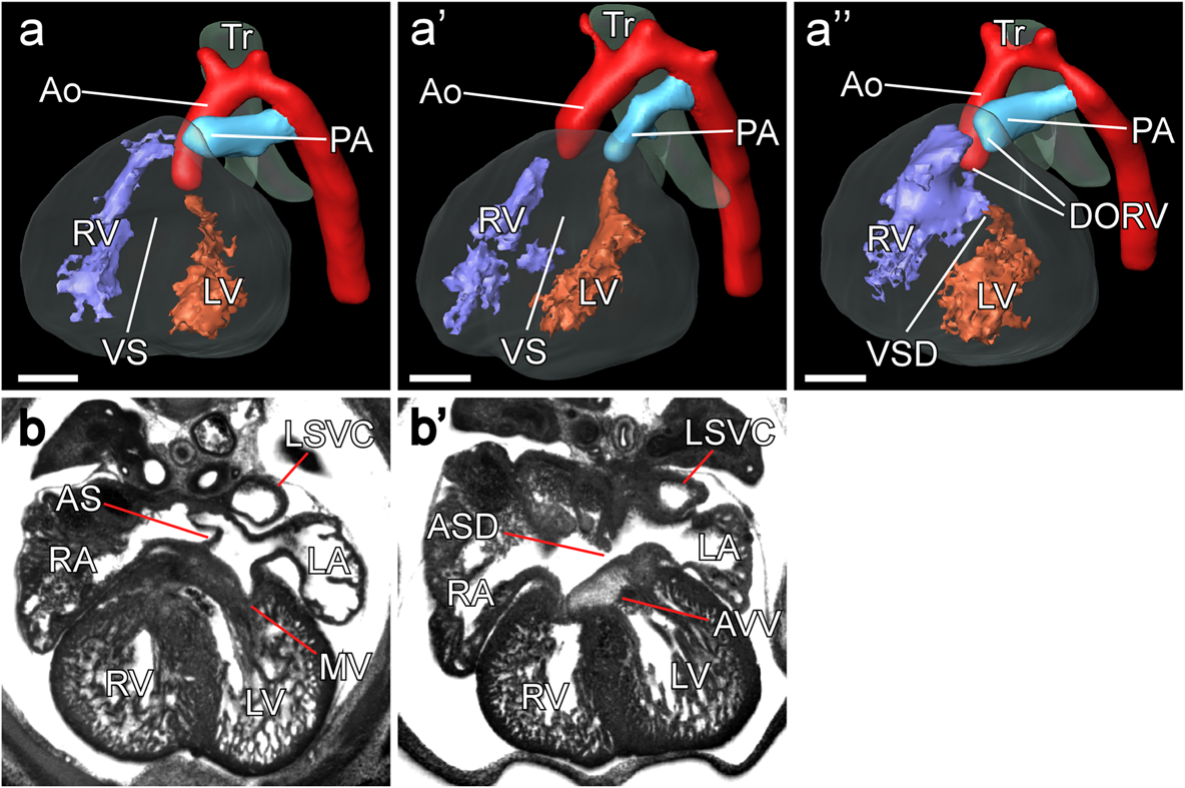
Cardiovascular anomalies in the 15.5dpc embryos with the *Mesp1Cre*-driven deletion of *Pcsk5*. (**a**) Three-dimensional reconstruction of a control (*Pcsk5* ^*wt/flox*^ *; Mesp1Cre*) heart. Left and right ventricles (*LV*, *RV*) are separated by ventricular septum (*VS*); the aorta (*Ao*) arises from the left ventricle and forms the arch on the left side of the trachea (*Tr*). Pulmonary artery (*PA*) leaves from the right ventricle. (*a’*, *a”*) Corresponding views of the two representative abnormal hearts (*Psck5* ^*Δ1/flox*^ *; Mesp1Cre*). (*a’*) Transposition of great arteries: aorta arises from the right, and pulmonary artery – from the left ventricle. (*a”*) Ventricular septal defect (*VSD*) and double outlet right ventricle (*DORV*): both great arteries arise from the right ventricle. (**b**) MRI section through the control heart. The mitral valve (*MV*) is formed between the left ventricle (*LV*) and the left atrium (*LA*). Atrial septum (*AS*) separates left and right atria (*RA*, *LA*). (*b’*) Corresponding sections through the heart of the *Psck5* ^*Δ1/flox*^ *; Mesp1Cre* embryo, showing abnormally formed, thickened atrioventricular valves (*AVV*) and atrial septal defect (*ASD*). Left superior vena cava (*LSVC*) is indicated in both sections. All scale bars = 0.5 mm

We next wished to determine the role of *Pcsk5* in mesodermal lineages that contribute to the heart, and we initially used a *Mesp1Cre* driver to investigate this. *Mesp1Cre* deletes in the extraembryonic and lateral mesoderm at the onset of gastrulation, at ~ E6.5 [31] (Table 2). Lateral mesodermal cells migrate through a primitive streak to the most anterior end of the embryo to become an anterior mesoderm that includes early cranio-cardiac and pharyngeal arch mesoderm [31, 32]. As evidenced by lineage tracing, this cell population contributes mainly to the mesoderm of the developing heart: myo-, endo- and epicardium [31, 33], cranial mesoderm-derived structures, like the muscles of the tongue, jaws and neck, and oesophagus [34, 35]. Other mesodermal lineages, paraxial (somites – bones, skeletal muscles), axial (notochord – neural tube) and intermediate (genitourinary tract, kidneys) are not affected by the *Mesp1*-driven Cre recombination [20, 31]. We analysed six *Pcsk5* ^*Δ1/flox*^ *; Mesp1Cre* ^*+*^ experimental embryos and six *Pcsk5* ^*wt/flox*^ *; Mesp1Cre* ^*+*^ control littermates. We observed atrial and ventricular septal defects, abnormal atrio-ventricular junction, double outlet right ventricle and transposition of great arteries in *Pcsk5* ^*Δ1/flox*^ *; Mesp1Cre* ^*+*^ embryos (Fig. 4, Table 1). Consistent with lack of deletion in paraxial, axial and intermediate mesoderm, there was no evidence of caudal regression, renal agenesis, or hindgut abnormalities. All control littermates were normal. These results showed that *Pcsk5* is required in the cranio-cardiac mesoderm for the development of the heart.

### Pcsk5 is not required in the Nkx2.5 expressing mesodermal lineage for cardiac or great vessel development

**Fig. 5:**
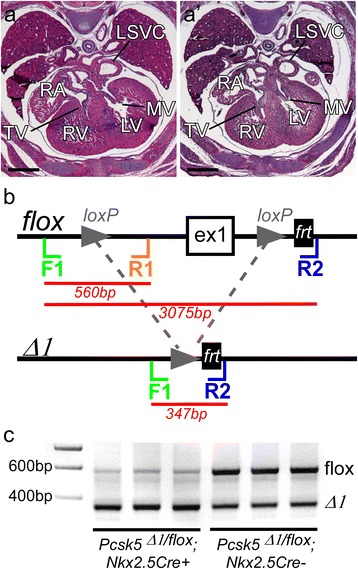
*Nkx2.5Cre*-driven deletion of *Pcsk5*. (**a**, *a’*) Transverse histological sections through the hearts of a control (*Psck5* ^*wt/flox*^ *; Nkx2.5Cre)* and *Psck5* ^*Δ1/flox*^ *; Nkx2.5Cre* embryos stained with hematoxylin and eosin. The mitral and tricuspid valves (*MV*, *TV*), right and left ventricles (*RV*, *LV*) atrium (RA) and left superior vena cava (*LSVC*) are indicated. Scale bars = 0.5 mm. (**b**) Structure (not to scale) of wildtype, floxed and conditionally deleted (*Δ1*) *Pcsk5* alleles, showing position of exon 1 (ex1), loxP and frt sites, and genotyping primers: F1, R1 and R2. Product sizes are indicated. The floxed allele has a diagnostic 560 nucleotide PCR product whereas the D1 allele has a diagnostic 347 nucleotide PCR product. (**c**) Ethidium bromide stained agarose gel showing multiplex polymerase chain reaction products of allele-specific genotyping from embryonic hearts. Three hearts from each genotype were analysed at E15.5. The floxed allele is almost completely absent in the hearts of *Cre*-positive *Pcsk5* ^*Δ1/flox*^ embryos

We next investigated the role of *Pcsk5* more specifically in cardiac progenitor lineages. To do this we used the *Nkx2.5Cre* driver [21]. This activates expression of Cre recombinase in the myocardial precursors of primary and secondary heart fields, with target floxed gene recombination effectively starting at embryonic day E7.75 [36] (Table 2). In addition, *Nkx2.5Cre* drives recombination also in the endoderm and ectoderm of the first pharyngeal arch [37]. Fate mapping shows that *Nkx2.5Cre* effectively deletes in the progenitor cells that contribute to myocardium, to coronary, chamber, and valve endothelium, to smooth muscle cells of the aorta and coronary arteries, to proepicardium and, subsequently, to coronary vasculature [38]. In keeping with this, *Nkx2.5Cre-*driven deletion of *Fgf8* results in a truncated heart tube and pharyngeal arch hypoplasia [38], and *Bmp4* (Bone morphogenetic protein 4) deletion leads to conotruncal and atrioventricular septation defects and anomalies in the branching of branchial arch arteries [39]. To explore the role of *Pcsk5* in the *Nkx2.5*-expressing lineage, we studied six *Pcsk5* ^*Δ1/flox*^ *; Nkx2.5Cre* ^*+*^ and six *Pcsk5* ^*wt/flox*^ *; Nkx2.5Cre* ^*+*^ control embryos. Surprisingly, no cardiac, outflow tract or pharyngeal arch malformations were observed (Fig. 5, Table 1). To investigate if *Nkx2.5Cre* was deleting *Pcsk5* from cardiac tissues, we used allele-specific polymerase chain reaction on the hearts obtained from these embryos. This showed that *Pcsk5* was indeed completely deleted in the heart (Fig. 5). Thus the cardiogenic progenitor deletion induced by *Mesp1Cre* is either not relevant to the development of the heart, or is needed at an earlier time point to be effective. Another possibility is that either mRNA or protein persisting from the earlier stages is sufficient to compensate for the deletion of *Pcsk5* with *Nkx2.5Cre* at the stage when this driver is active. Alternatively, another member of the convertase family may effectively substitute for the function of PCSK5 in these tissues.

Although *Nkx2.5Cre*-driven deletion of *Pcsk5* did not lead to cardiac developmental anomalies, tissues derived from the heart fields may still require the expression of *Pcsk5*; the nature of atrioventricular septal defect observed in *Pcsk5* knock-out embryos (Figs. 2b’ and 4b’) may suggest developmental anomalies in the formation of dorsal mesenchymal protrusion (DMP). DMP is a derivative of the posterior second heart field (SHF) and is critical for the formation of the atrioventricular mesenchymal complex [40]. It provides a mesenchymal “connector” between the major atrioventricular cushions and the cap of the primary atrial septum (*septum primum*), and after myocardial differentiation, it transforms into a muscular rim at the base of the atrial septum [40]. Mice with mutations (or deletions) in the genes important for posterior SHF, like *Alk3*, *Pdpn* or *Tbx5* show atrioventricular septal defects similar to this observed in the *Pcsk5* mutants [41, 42]. Moreover, the development of DMP is, among others, regulated by BMP signalling [40] and members of BMP family are potential substrates for *Pcsk5*. Testing the requirements for *Pcsk5* in the SHF (and subsequently in DMP), using, for example, a *Mef2cCre* driver, would be the next logical step and will require further investigation.

### Pcsk5 is not required in the pharyngeal arches for cardiac or great vessel development

Pharyngeal arch tissues initially include all three germ layers: endo-, meso- and ectoderm, and are subsequently populated by neural crest cells [43]. The pharyngeal arches contribute to several cranio-facial structures like bones, muscles and nerves. They also give rise to the aorta and great vessels [43]. To ablate *Pcsk5* in pharyngeal arch tissues we used the *Hoxa3Cre* driver [22]. *Hoxa3Cre*-dependent recombination initiates weakly just before E8.0 and by E9.0 affects all pharyngeal tissues caudal to the second arch [44] (Table 2). In keeping with this, deletion of *Tbx1* (T-box transcription factor 1) in pharyngeal arch tissues, for instance, results in aortic arch malformations, including right-sided aortic arch, aortic vascular ring, and outflow tract septation defects [44, 45]. To explore the role of *Pcsk5* in the *Hoxa3-*expressing pharyngeal arch lineages, we analysed five *Pcsk5* ^*Δ1/flox*^ *; Hoxa3Cre* ^*+*^ and five control littermate embryos. We did not observe any cardiac or outflow tract anomalies. There were also no other obvious structural defects, detectable by MRI, in these embryos (Table 1). To investigate if *Hoxa3Cre* was deleting *Pcsk5*, we used allele-specific polymerase chain reaction on the hearts obtained from these embryos. This showed that *Pcsk5* was indeed deleted in the pharyngeal arches (Additional file 1: Figure S1). This result indicates that the expression of *Pcsk5* is not essential in the ecto-, meso- and endodermal pharyngeal arch lineages during embryonic development. Thus, the pharyngeal mesodermal deletion induced by *Mesp1-Cre* is either not relevant to the development of the aortic arches, or is needed at an earlier time point to be effective.

### Deletion of Pcsk5 in the neural crest did not affect heart development

The neural crest originates in the ectoderm, and contributes to the outflow tract of the heart, providing cells and mediating remodelling of the cardiac outflow and aortic arches. Neural crest cells also form smooth muscle and innervate the cardiovascular system [46]. *Wnt1Cre* deletes specifically in the neural crest, and labelled cells contribute to the aorticopulmonary septum and cono-truncal cushions, and to the 3^rd^, 4^th^ and 6^th^ pharyngeal arch arteries [15] (Table 2). In keeping with this, *Wnt1Cre*-driven deletion of *Ptpn11* (Protein tyrosine phosphatase, non-receptor type 11) results in common arterial trunk and abnormal great vessels [47], deletion of *Mapk1* (Mitogen-activated protein kinase 1) – in double outlet right ventricle and septal defects [48], and deletion of *Acvr1* (Activin A receptor, type I) - in common arterial trunk, septal defects and anomalies in brachiocephalic arteries [49]. The zygotic mutation of *Pcsk5* and *Sox2Cre* conditional knockout shows common arterial trunk as a phenotype, and this was not observed in mesodermal deletion of *Pcsk5* with *Mesp1Cre* (Table 1). As common arterial trunk is a neural crest phenotype [50], and is also observed in conditional deletions of different genes induced by *Wnt1Cre*, we explored the role of *Pcsk5* in neural crest development. We examined eight *Pcsk5* ^*Δ1/flox*^ *; Wnt1Cre* ^*+*^ and ten control *Pcsk5* ^*wt/flox*^ *; Wnt1Cre* ^*+*^ embryos. We did not see any developmental anomalies in examined embryos (Table 1). This indicates that *Pcsk5* is not required in the neural crest during cardiac development.

## Conclusions

In summary, our results show that although *Pcsk5* is expressed in the heart and outflow tract and pharyngeal arches at later developmental stages, for normal heart, outflow tract and aortic arch development only its deletion in the cranio-cardiac mesoderm appears to have an effect on heart development. These results suggest that *Pcsk5* may have an essential and early role in the cranio-cardiac mesoderm for heart development. Alternatively, it is possible that *Pcsk5* may still play a critical role in *Nkx2.5*-expressing cardiac progenitors, with persistence of mRNA or protein accounting for the lack of effect of deletion on heart development. Our studies thus define a window of development during which epigenetic factors for instance may interact with heterozygous *Pcsk5*/*PCSK5* mutations to affect heart development, and may explain variable penetrance of phenotype observed in humans with *PCSK5* mutation [1, 5].

## Additional file

Additional file 1: Figure S1.
*Hoxa3Cre*-driven deletion of *Pcsk5*. Ethidium bromide stained agarose gel showing multiplex polymerase chain reaction products of allele-specific genotyping from embryonic hearts. Primer details are as in Fig. 5. Five hearts of the *Pcsk5* ^*Δ1/flox*^ *; Hoxa3Cre* ^*+*^ embryos were analysed. The floxed allele is almost completely absent in this hearts indicating a loss of *Pcsk5* floxed allele. (TIF 477 kb)

## Abbreviations

Acvr1: Activin A receptor, type I
Alk3: Bone morphogenetic protein receptor, type 1A (Bmpr1a)
ASD: Atrial septal defect
BMP/Bmp: Bone morphogenetic protein
CAT: Common arterial trunk
CHD: Congenital heart disease
DMP: Dorsal mesenchymal protrusion
E: Embryonic day
Fgf8: Fibroblast growth factor 8
GDF11: Growth differentiation factor 11
Hox: Homeobox
KO: Knock-out
Mapk1: Mitogen-Activated Protein Kinase 1
Mef2c: Myocyte Enhancer Factor 2C)
Meox2: Mesenchyme Homeobox 2
Mesp1: Mesoderm Posterior BHLH Transcription Factor 1
MRI: Magnetic resonance imaging
mRNA: Messenger ribonucleic acid
Nkx2.5: NK2 Homeobox 5
PBS: Phosphate buffered saline
PCSK5 / Pcsk5: Proprotein convertase subtilisin/kexin type 5
Pdpn: Podoplanin
Ptpn11: Protein tyrosine phosphatase, non-receptor type 11
SHF: Second heart field
Sox2: SRY (sex determining region Y)-box 2
Tbx1: T-box transcription factor 1
Tbx5: T-box transcription factor
Tgfβ: Transforming growth factor beta
VACTERL: Vertebral anomalies, Anal atresia, Cardiac defects, Tracheoesophageal fistula and/or Esophageal atresia, Renal & Radial anomalies and Limb defects
Vcc: Ethylnitrosourea-induced *Pcsk5* mutation
VSD: Ventricular septal defect

## References

[1] Szumska D, Pieles G, Essalmani R, Bilski M, Mesnard D, Kaur K, Franklyn A, El Omari K, Jefferis J, Bentham J (2008). VACTERL/caudal regression/Currarino syndrome-like malformations in mice with mutation in the proprotein convertase Pcsk5. Genes Dev.

[2] Essalmani R, Zaid A, Marcinkiewicz J, Chamberland A, Pasquato A, Seidah NG, Prat A (2008). In vivo functions of the proprotein convertase PC5/6 during mouse development: Gdf11 is a likely substrate. Proc Natl Acad Sci U S A.

[3] Clark EB (2004). Evolution, genetics, and the etiology of congenital cardiovascular malformations. J Pediatr.

[4] Hoffman JI, Kaplan S (2002). The incidence of congenital heart disease. J Am Coll Cardiol.

[5] Nakamura Y, Kikugawa S, Seki S, Takahata M, Iwasaki N, Terai H, Matsubara M, Fujioka F, Inagaki H, Kobayashi T (2015). PCSK5 mutation in a patient with the VACTERL association. BMC Res Notes.

[6] Seidah NG, Prat A (2012). The biology and therapeutic targeting of the proprotein convertases. Nat Rev Drug Discov.

[7] Mesnard D, Constam DB (2010). Imaging proprotein convertase activities and their regulation in the implanting mouse blastocyst. J Cell Biol.

[8] Essalmani R, Hamelin J, Marcinkiewicz J, Chamberland A, Mbikay M, Chretien M, Seidah NG, Prat A (2006). Deletion of the gene encoding proprotein convertase 5/6 causes early embryonic lethality in the mouse. Mol Cell Biol.

[9] Moorman AF, Christoffels VM (2003). Cardiac chamber formation: development, genes, and evolution. Physiol Rev.

[10] Tam PP, Gad JM, Stern CD (2004). Gastrulation in the mouse embryo. Gastrulation: from cells to embryo.

[11] Harvey RP (2002). Patterning the vertebrate heart. Nat Rev Genet.

[12] Kelly RG, Buckingham ME (2002). The anterior heart-forming field: voyage to the arterial pole of the heart. Trends Genet.

[13] Solloway MJ, Harvey RP (2003). Molecular pathways in myocardial development: a stem cell perspective. Cardiovasc Res.

[14] Cai CL, Liang X, Shi Y, Chu PH, Pfaff SL, Chen J, Evans S (2003). Isl1 identifies a cardiac progenitor population that proliferates prior to differentiation and contributes a majority of cells to the heart. Dev Cell.

[15] Jiang X, Rowitch DH, Soriano P, McMahon AP, Sucov HM (2000). Fate of the mammalian cardiac neural crest. Development.

[16] de Lange FJ, Moorman AF, Anderson RH, Manner J, Soufan AT, de Gier-de Vries C, Schneider MD, Webb S, van den Hoff MJ, Christoffels VM (2004). Lineage and morphogenetic analysis of the cardiac valves. Circ Res.

[17] Hiruma T, Nakajima Y, Nakamura H (2002). Development of pharyngeal arch arteries in early mouse embryo. J Anat.

[18] Xin M, Olson EN, Bassel-Duby R (2013). Mending broken hearts: cardiac development as a basis for adult heart regeneration and repair. Nat Rev Mol Cell Biol.

[19] Hayashi S, Lewis P, Pevny L, McMahon AP (2002). Efficient gene modulation in mouse epiblast using a Sox2Cre transgenic mouse strain. Mech Dev.

[20] Saga Y, Kitajima S, Miyagawa-Tomita S (2000). Mesp1 expression is the earliest sign of cardiovascular development. Trends Cardiovasc Med.

[21] Moses KA, DeMayo F, Braun RM, Reecy JL, Schwartz RJ (2001). Embryonic expression of an Nkx2-5/Cre gene using ROSA26 reporter mice. Genesis.

[22] Macatee TL, Hammond BP, Arenkiel BR, Francis L, Frank DU, Moon AM (2003). Ablation of specific expression domains reveals discrete functions of ectoderm- and endoderm-derived FGF8 during cardiovascular and pharyngeal development. Development.

[23] Hayashi S, Tenzen T, McMahon AP (2003). Maternal inheritance of Cre activity in a Sox2Cre deleter strain. Genesis.

[24] Schneider JE, Bose J, Bamforth SD, Gruber AD, Broadbent C, Clarke K, Neubauer S, Lengeling A, Bhattacharya S (2004). Identification of cardiac malformations in mice lacking Ptdsr using a novel high-throughput magnetic resonance imaging technique. BMC Dev Biol.

[25] Bamforth SD, Braganca J, Eloranta JJ, Murdoch JN, Marques FI, Kranc KR, Farza H, Henderson DJ, Hurst HC, Bhattacharya S (2001). Cardiac malformations, adrenal agenesis, neural crest defects and exencephaly in mice lacking Cited2, a new Tfap2 co-activator. Nat Genet.

[26] Moore R, Cai KQ, Tao W, Smith ER, Xu XX (2013). Differential requirement for Dab2 in the development of embryonic and extra-embryonic tissues. BMC Dev Biol.

[27] Barak Y, Nelson MC, Ong ES, Jones YZ, Ruiz-Lozano P, Chien KR, Koder A, Evans RM (1999). PPAR gamma is required for placental, cardiac, and adipose tissue development. Mol Cell.

[28] Avilion AA, Nicolis SK, Pevny LH, Perez L, Vivian N, Lovell-Badge R (2003). Multipotent cell lineages in early mouse development depend on SOX2 function. Genes Dev.

[29] Maruyama EO, Lin H, Chiu SY, Yu HM, Porter GA, Hsu W (2016). Extraembryonic but not embryonic SUMO-specific protease 2 is required for heart development. Sci Rep.

[30] Zhu D, Holz S, Metzger E, Pavlovic M, Jandausch A, Jilg C, Galgoczy P, Herz C, Moser M, Metzger D (2014). Lysine-specific demethylase 1 regulates differentiation onset and migration of trophoblast stem cells. Nat Commun.

[31] Saga Y, Miyagawa-Tomita S, Takagi A, Kitajima S, Miyazaki J, Inoue T (1999). MesP1 is expressed in the heart precursor cells and required for the formation of a single heart tube. Development.

[32] Watanabe Y, Miyagawa-Tomita S, Vincent SD, Kelly RG, Moon AM, Buckingham ME (2010). Role of mesodermal FGF8 and FGF10 overlaps in the development of the arterial pole of the heart and pharyngeal arch arteries. Circ Res.

[33] Kang J, Gu Y, Li P, Johnson BL, Sucov HM, Thomas PS (2008). PDGF-A as an epicardial mitogen during heart development. Dev Dyn.

[34] Gopalakrishnan S, Comai G, Sambasivan R, Francou A, Kelly RG, Tajbakhsh S (2015). A cranial mesoderm origin for esophagus striated muscles. Dev Cell.

[35] Bildsoe H, Loebel DA, Jones VJ, Hor AC, Braithwaite AW, Chen YT, Behringer RR, Tam PP (2013). The mesenchymal architecture of the cranial mesoderm of mouse embryos is disrupted by the loss of Twist1 function. Dev Biol.

[36] Stanley EG, Biben C, Elefanty A, Barnett L, Koentgen F, Robb L, Harvey RP (2002). Efficient Cre-mediated deletion in cardiac progenitor cells conferred by a 3′UTR-ires-Cre allele of the homeobox gene Nkx2-5. Int J Dev Biol.

[37] Ilagan R, Abu-Issa R, Brown D, Yang YP, Jiao K, Schwartz RJ, Klingensmith J, Meyers EN (2006). Fgf8 is required for anterior heart field development. Development.

[38] Ma Q, Zhou B, Pu WT (2008). Reassessment of Isl1 and Nkx2-5 cardiac fate maps using a Gata4-based reporter of Cre activity. Dev Biol.

[39] Liu W, Selever J, Wang D, Lu MF, Moses KA, Schwartz RJ, Martin JF (2004). Bmp4 signaling is required for outflow-tract septation and branchial-arch artery remodeling. Proc Natl Acad Sci U S A.

[40] Burns T, Yang Y, Hiriart E, Wessels A. The dorsal mesenchymal protrusion and the pathogenesis of atrioventricular septal defects. J Cardiovasc Dev Dis. 2016;3(4). doi: 10.3390/jcdd3040029. Epub 2016 Sep 26. 10.3390/jcdd3040029 PMC5267359 28133602

[41] Briggs LE, Phelps AL, Brown E, Kakarla J, Anderson RH, van den Hoff MJ, Wessels A (2013). Expression of the BMP receptor Alk3 in the second heart field is essential for development of the dorsal mesenchymal protrusion and atrioventricular septation. Circ Res.

[42] Douglas YL, Mahtab EA, Jongbloed MR, Uhrin P, Zaujec J, Binder BR, Schalij MJ, Poelmann RE, Deruiter MC, Gittenberger-de Groot AC (2009). Pulmonary vein, dorsal atrial wall and atrial septum abnormalities in podoplanin knockout mice with disturbed posterior heart field contribution. Pediatr Res.

[43] Graham A (2003). Development of the pharyngeal arches. Am J Med Genet A.

[44] Zhang Z, Cerrato F, Xu H, Vitelli F, Morishima M, Vincentz J, Furuta Y, Ma L, Martin JF, Baldini A (2005). Tbx1 expression in pharyngeal epithelia is necessary for pharyngeal arch artery development. Development.

[45] Vitelli F, Zhang Z, Huynh T, Sobotka A, Mupo A, Baldini A (2006). Fgf8 expression in the Tbx1 domain causes skeletal abnormalities and modifies the aortic arch but not the outflow tract phenotype of Tbx1 mutants. Dev Biol.

[46] Brown CB, Baldwin HS (2006). Neural crest contribution to the cardiovascular system. Adv Exp Med Biol.

[47] Nakamura T, Gulick J, Colbert MC, Robbins J (2009). Protein tyrosine phosphatase activity in the neural crest is essential for normal heart and skull development. Proc Natl Acad Sci U S A.

[48] Newbern J, Zhong J, Wickramasinghe RS, Li X, Wu Y, Samuels I, Cherosky N, Karlo JC, O’Loughlin B, Wikenheiser J (2008). Mouse and human phenotypes indicate a critical conserved role for ERK2 signaling in neural crest development. Proc Natl Acad Sci U S A.

[49] Kaartinen V, Dudas M, Nagy A, Sridurongrit S, Lu MM, Epstein JA (2004). Cardiac outflow tract defects in mice lacking ALK2 in neural crest cells. Development.

[50] Hutson MR, Kirby ML (2003). Neural crest and cardiovascular development: a 20-year perspective. Birth Defects Res C Embryo Today.

[51] Danielian PS, Muccino D, Rowitch DH, Michael SK, McMahon AP (1998). Modification of gene activity in mouse embryos in utero by a tamoxifen-inducible form of Cre recombinase. Curr Biol.

[52] Bertrand N, Roux M, Ryckebusch L, Niederreither K, Dolle P, Moon A, Capecchi M, Zaffran S (2011). Hox genes define distinct progenitor sub-domains within the second heart field. Dev Biol.

