# Supplementary figures and images for: Pcsk5 is required in the early cranio-cardiac mesoderm for heart development
Source: BMC Dev Biol. 2017 Apr 26;17:6. doi: 10.1186/s12861-017-0148-y (PMC5407003; doi:10.1186/s12861-017-0148-y)

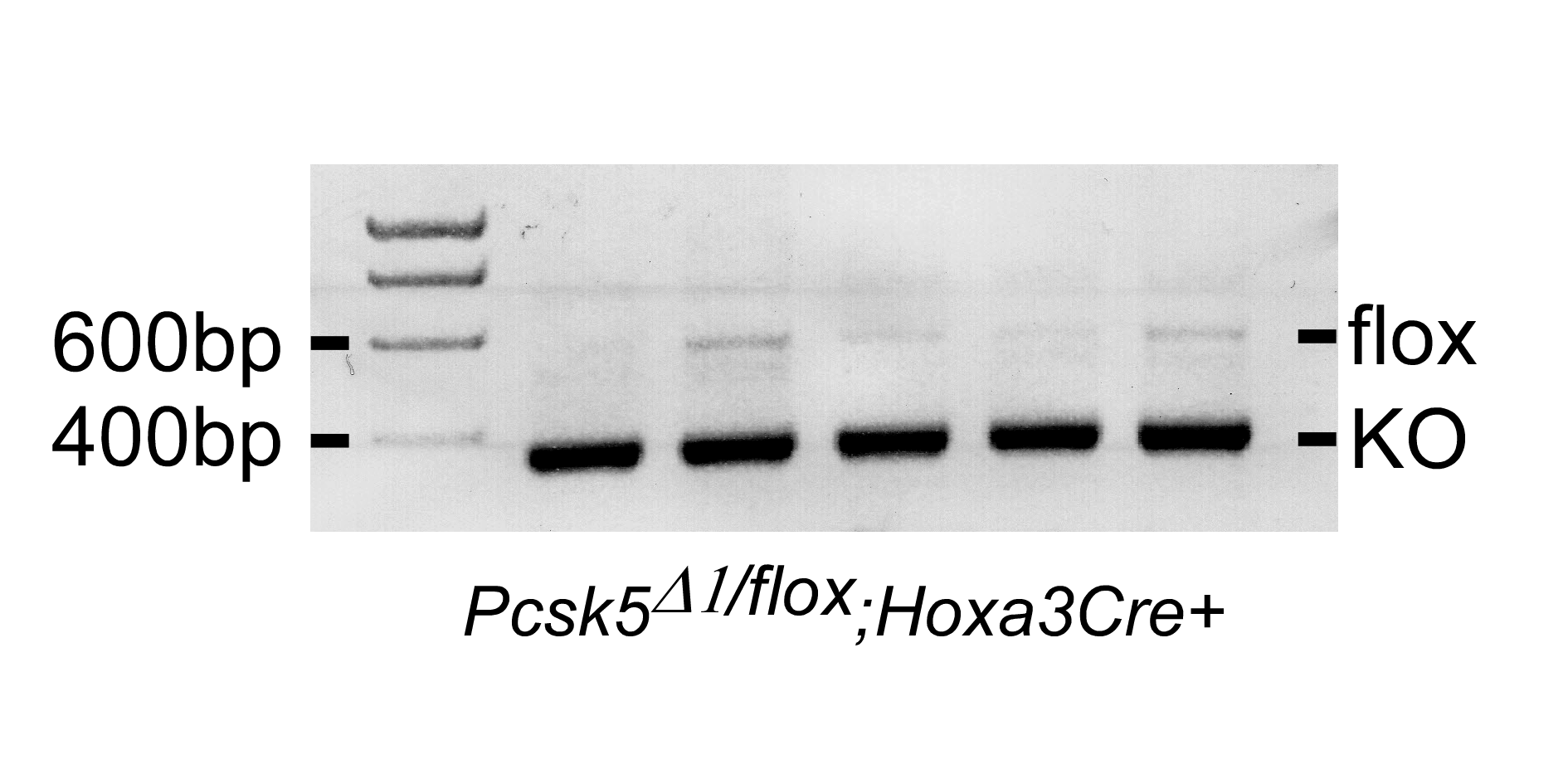

Supplement: Additional file 1: Figure S1. — Hoxa3Cre-driven deletion of Pcsk5. Ethidium bromide stained agarose gel showing multiplex polymerase chain reaction products of allele-specific genotyping from embryonic hearts. Primer details are as in Fig. 5. Five hearts of the Pcsk5 Δ1/flox ; Hoxa3Cre + embryos were analysed. The floxed allele is almost completely absent in this hearts indicating a loss of Pcsk5 floxed allele. (TIF 477 kb) [file 12861_2017_148_MOESM1_ESM.tif]
